# NMR Analysis of the Conformational Properties of the Trapped on-pathway Folding Intermediate of the Bacterial Immunity Protein Im7

**DOI:** 10.1016/j.jmb.2006.11.012

**Published:** 2007-02-23

**Authors:** Sara B.-M. Whittaker, Graham R. Spence, J. Günter Grossmann, Sheena E. Radford, Geoffrey R. Moore

**Affiliations:** 1 School of Chemical Sciences and Pharmacy, University of East Anglia, Norwich NR4 7TJ, UK; 2 Astbury Centre for Structural Molecular Biology, University of Leeds, Leeds LS2 9JT, UK; 3 Molecular Biophysics Group, CLRC Daresbury Laboratory, Daresbury, Warrington WA4 4AD, UK

**Keywords:** HSQC, heteronuclear single quantum coherence, Im7, the immunity protein for colicin E7, MD, molecular dynamics, NOE, nuclear Overhauser enhancement, NOESY, NOE spectroscopy, SIS, stable intermediate state, KIS, kinetic intermediate state, EIS, exchange-competent intermediate state, SAXS, small-angle X-ray scattering, protein folding, intermediate, NMR, SAXS, Im7

## Abstract

Previous work shows that the transiently populated, on-pathway intermediate in Im7 folding contains three of the four native α-helices docked around a core stabilised by native and non-native interactions. To determine the structure and dynamic properties of this species in more detail, we have used protein engineering to trap the intermediate at equilibrium and analysed the resulting proteins using NMR spectroscopy and small angle X-ray scattering. Four variants were created. In L53AI54A, two hydrophobic residues within helix III are truncated, preventing helix III from docking stably onto the developing hydrophobic core. In two other variants, the six residues encompassing the native helix III were replaced with three (H3G3) or six (H3G6) glycine residues. In the fourth variant, YY, two native tyrosine residues (Tyr55 and Tyr56) were re-introduced into H3G6 to examine their role in determining the properties of the intermediate ensemble. All four variants show variable peak intensities and broad peak widths, consistent with these proteins being conformationally dynamic. Chemical shift analyses demonstrated that L53AI54A and YY contain native-like secondary structure in helices I and IV, while helix II is partly formed and helix III is absent. Lack of NOEs and rapid NH exchange for L53AI54A, combined with detailed analysis of the backbone dynamics, indicated that the hydrophobic core of this variant is not uniquely structured, but fluctuates on the NMR timescale. The results demonstrate that though much of the native-like secondary structure of Im7 is present in the variants, their hydrophobic cores remain relatively fluid. The comparison of H3G3/H3G6 and L53AI54A/YY suggests that Tyr55 and/or Tyr56 interact with the three-helix core, leading other residues in this region of the protein to dock with the core as folding progresses. In this respect, the three-helix bundle acts as a template for formation of helix III and the creation of the native fold.

## Introduction

Though many single-domain proteins fold in two-state transitions from their unfolded to folded states,^1–3^ some have been shown to fold *via* partially folded intermediates.^4–10^ In principle, intermediates between the unfolded and folded states may enhance the rate of folding by reducing the conformational space through which the polypeptide chain has to search, or may retard the rate of folding by sequestering the polypeptide chain in a stable, partially folded state.^11^^,^^12^ Such energetic traps have been shown, most often, to involve species that have a native-like topology stabilised by a subset of the native contacts, and in some cases, by significant non-native interactions.^8–10^^,^^13^ Determining the conformational properties of intermediate states at as high a resolution as possible is therefore important for a full elucidation of the structural mechanism of folding. This poses a significant experimental challenge, however, as a consequence of the transient nature of intermediate states, which generally means they are present in low concentrations relative to the unfolded and/or folded states, and from their conformational dynamics. Insight into the conformational properties of intermediates is now becoming clear using a variety of NMR methods, including relaxation dispersion experiments, which, providing that the conformational exchange occurs with the native state on a suitable timescale, can reveal the chemical shifts of resonances of species populated transiently and only rarely (e.g. to 1%).^14^ NMR analysis of proteins trapped in a partially folded state, either by alteration of the solution conditions,^15–17^ or by mutagenesis to create a sequence in which the partially folded state is the lowest energy species, have also provided insights into the nature of these ensembles.^9^^,^^10^^,^^18^ Here we report NMR studies of the bacterial immunity protein Im7, and variants of it constructed to trap its on-pathway folding intermediate at equilibrium.

**Figure 1:**
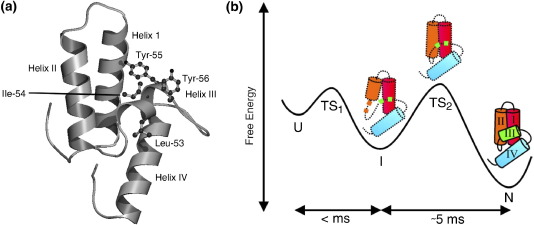
(a) Cartoon of the structure of Im7 (pdb:1ayi)^20^ constructed with Molscript.^21^ The side-chains of important helix III residues, Leu53, Ile54, Tyr55 and Tyr56, are shown. (b) Schematic diagram of the folding mechanism of Im7^⁎^. The four helices of native Im7 are coloured differently. The scheme highlights that this small, single-domain protein folds *via* a three-helical intermediate and rate-limiting transition state, that differ from each other in the packing of the helices. Intermediate formation occurs on the sub-milliseconds timescale. Data presented in this article indicate that the C-terminal region of helix II is not uniquely formed in the intermediate ensemble, but remains conformationally dynamic at this stage of folding.

Im7 is a 9.5 kDa inhibitor of the bacterial colicin DNase E7, which provides immunity against the lethal action of the colicin to the producing *Escherichia coli* cell.^19^ Im7 adopts a distorted four-helix bundle structure, in which helices I and II form a hairpin, with helix IV and the shorter helix III packed across its face (Figure 1(a)).^20^ The small size of Im7, its single tryptophan residue, and lack of disulfide bonds, prosthetic groups and *cis*-proline residues in the native state, make it a good subject for protein folding studies. We have shown that Im7 folds by a three-state transition at pH 7.0, *via* a transiently populated on-pathway kinetic intermediate state (KIS), which is hyper-fluorescent (Figure 1(b)).^4^^,^^22^ φ-Value analysis has indicated that the KIS of Im7^⁎^ (the ⁎ indicating an N-terminal His-tag) is a compact species (β_T_ = 0.75) that contains helical regions corresponding to helices I, II and IV of native Im7, but lacks regular secondary structure in the sequence corresponding to the native helix III.^13^ Measurement of equilibrium NH exchange rates demonstrated that the exchange-competent intermediate state of Im7^⁎^ (EIS) also contains helices I, II and IV and lacks helix III.^23^ Subsequent molecular dynamics simulations using the NH exchange protection values as restraints showed that the intermediate is a three-helical bundle species stabilised by a hydrophobic core involving both native and non-native interactions.^24^

Spence *et al.*^25^ adopted a different approach for investigating the Im7 folding intermediate by constructing a series of variant proteins designed to trap Im7^⁎^ in its intermediate state by selectively destabilising the native state. The stable intermediate state (SIS) was generated by preventing helix III from docking onto the core structure containing helices I, II and IV using two approaches (summarised in Table 1). First, key interactions were removed by substituting the side-chains of Leu53 and Ile54 with alanine, creating the variant L53AI54A. The second approach involved replacing the six residues comprising the native helix III of Im7^⁎^ with either a three-residue or a six-residue polyglycine sequence, creating the variants H3G3 and H3G6, respectively.^25^ Previous work^25^ has shown that these variants have similar thermodynamic (Δ*G*_UF_ ∼10 kJ/mol) and biophysical properties (compactness and secondary structural content) to the kinetic intermediate. However, the fluorescence properties of the H3G3 and H3G6 variants differ significantly from those of the kinetic intermediate and the variant L53AI54A, the latter two having unusual and characteristic hyper-fluorescent signals, suggesting that the environment of the single tryptophan (Trp75) differs in the different variants. To investigate this further, a fourth variant, YY, was constructed in which the two native tyrosine residues (Tyr55 and Tyr56) were put back into the H3G6 background, restoring the hyper-fluorescence properties of the KIS and the variant L53AI54A.^26^ Ultra-violet Resonance Raman studies^26^ indicated that in the trapped intermediate species the environment of Trp75 is more hydrophobic than in wild-type Im7^⁎^, confirming a conformational link between the tryptophan environment and residues in helix III, even though these regions are far apart in the native structure.^20^

Here we report studies of the structural and dynamic properties of native Im7^⁎^ and the SIS variants L53AI54A, YY, H3G3 and H3G6 using multi-nuclear NMR methods combined with small-angle X-ray scattering (SAXS), with the aim of defining the structural and dynamic properties of the trapped on-pathway folding intermediate of this small, single-domain protein at the residue-specific level in as much detail as possible.

## Results and Discussion

### ^1^H-^15^N HSQC NMR spectra of Im7⁎ and its SIS variants

**Figure 2:**
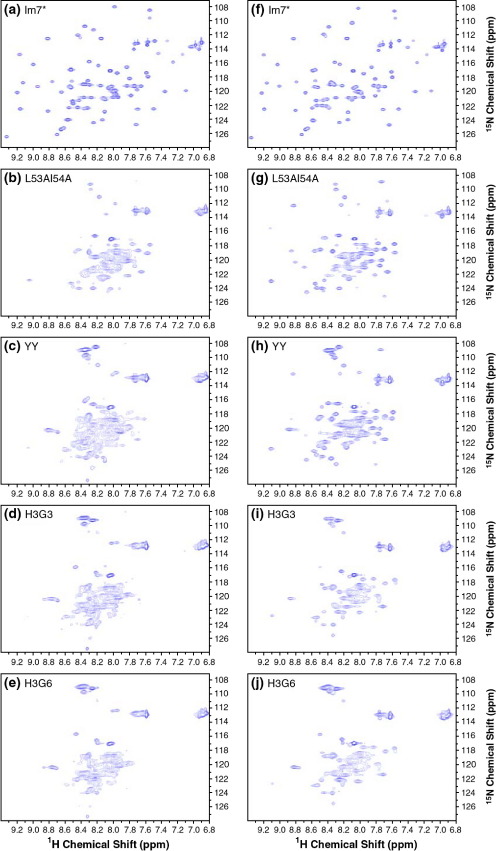
Effect of sodium sulphate on ^1^H-^15^N HSQC spectra of wild-type Im7^⁎^ and its variants. The absence (left panels) and presence (right panels) of 400 mM sodium sulphate is shown for (a) and (f) wild-type Im7^⁎^, (b) and (g) L53AI54A, (c) and (h) YY, (d) and (i) H3G3 and (e) and (j) H3G6. All spectra were measured at 500 MHz, 298 K on samples containing ∼1 mM protein concentration in 50 mM sodium phosphate buffer (pH 7.0), 90% H_2_O/10% ^2^H_2_O.

^1^H-^15^N heteronuclear single quantum coherence (HSQC) NMR spectra of Im7^⁎^ and its SIS variants were measured to determine the optimum solution conditions for characterising the conformational properties of these proteins. Poor quality spectra were obtained for all of the SIS variants in 50 mM phosphate buffer (pH 7) (Figure 2(b)–(e)). However, upon the addition of 400 mM sodium sulphate the quality of the spectra was considerably improved (Figure 2(g)–(j)). Increasing the sodium sulphate concentration beyond 400 mM did not improve the appearance of the spectra further. It is notable that the spectrum of Im7^⁎^ was not affected by the addition of this kosmotrope (Figure 2(a) and (f)). Equilibrium analytical ultracentrifugation showed that Im7^20^ and the SIS variants^25^ are monomeric at the concentrations used for the NMR analysis. Furthermore, ^1^H-^15^N HSQC NMR spectra of Im7^⁎^ and the SIS variants were indistinguishable in terms of the numbers of peaks, their linewidths and apparent intensities over the concentration range 0.3–1.0 mM (data not shown).

The dispersion of resonances in the ^1^H-^15^N HSQC NMR spectra of Im7^⁎^ and its SIS variants in 400 mM sodium sulphate reveals that all the SIS proteins are at least partially structured under these conditions. However, there are notable differences between the spectra of the different proteins, both in terms of resonance chemical shifts and linewidths, with the variants falling into two groups: L53AI54A and YY showing greater chemical shift dispersion and narrower linewidths compared with H3G3 and H3G6 (Figure 2(g)–(j)). Previous studies of the effect of sodium sulphate on the folding of Im7^⁎^ have shown that this kosmotrope stabilises the kinetic intermediate without making it more compact, as judged by *M*-value analysis,^27^ suggesting that the reduction in resonance linewidths results from alterations of the rates of exchange within the ensemble rather than gross structural changes. The superior nature of the NMR spectra of L53AI54A and YY compared with those of H3G3 and H3G6, together with the observation by Spence *et al.*^25^ that the former are hyper-fluorescent like the KIS while the latter are not, led us to restrict further detailed NMR analysis to L53AI54A and YY.

### Global structural properties of Im7, Im7⁎ and the SIS variants

The hydrodynamic radii (*R*_h_) of Im7 (wild-type, no His-tag), Im7^⁎^ (wild-type plus His-tag) and the SIS variants (all of which have N-terminal His-tags) were determined by a PFG-NMR procedure.^28^ Table 1 summarises the mean values of *R*_h_ for each of the proteins along with the compaction factors (*C*) derived from these radii. The data confirm that all of the SIS variants are compact species, having *R*_h_ and *C* values that differ by less than 26% compared with their wild-type counterpart, Im7^⁎^. The His-tag reduces considerably the derived *C* values for these proteins as revealed by the comparison of the data for Im7 and Im7^⁎^ (*C* = 1.04 and 0.87, respectively). It is striking that H3G3 and H3G6 have a greater *R*_h_ than the other variants studied, and correspondingly lower compaction than Im7^⁎^, whilst L53AI54A is the most compact of all the variants studied, with an *R*_h_ indistinguishable from that of wild-type Im7^⁎^ (Table 1). The increase in compaction of L53AI54A compared with H3G3 and H3G6 mirrors the magnitude of *M*_UF_ values determined for these proteins using urea denaturation.^25^

**Figure 3:**
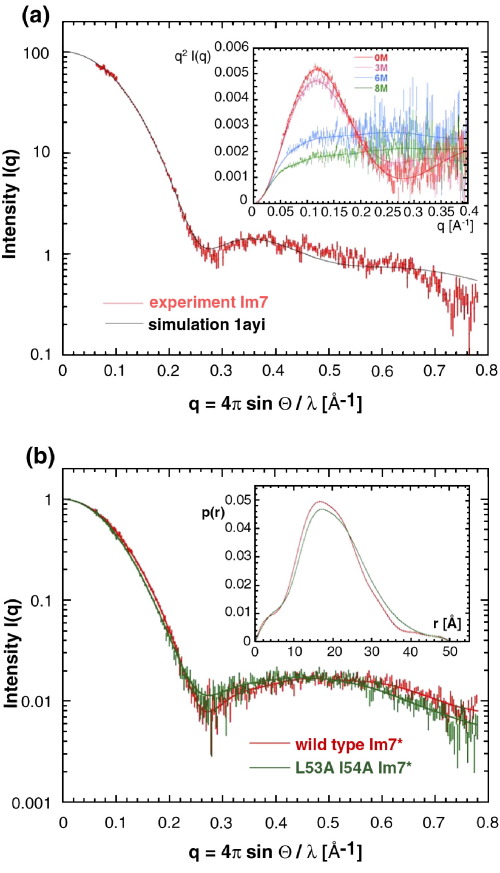
(a) X-ray scattering profile of Im7 in solution. Experimental data points are shown in red. The smooth curve represents the theoretical scattering profile based on the crystal structure of Im7 (pdb:1ayi).^20^ The inset depicts the Kratky plot of native Im7 compared with the protein in the presence of 0, 3, 6 or 8 M urea derived from the solution X-ray scattering data recorded at 10 °C. (b) Solution X-ray scattering profiles of Im7^⁎^ and the L53AI54A variant, measured at 10 °C in the presence of 400 mM sodium sulphate. The inset shows the experimental distance distribution functions *p*(*r*) for both proteins.

Additional information about the shape and size of a protein in solution can be obtained using SAXS. The SAXS profile of a globular protein reflects its structure, as demonstrated, for instance, by the Kratky plot for the addition of urea to Im7 (inset in Figure 3(a)). Figure 3(a) shows the scattering profile recorded for Im7 superimposed on the scattering curve calculated from the X-ray structure. A remarkable agreement is seen over the whole scattering range (indicated by a goodness-of-fit value, χ, of 2.05), which signifies that the solution structure of Im7 is well described by the crystal structure. Figure 3(b) compares the scattering profiles for Im7^⁎^ and the SIS variant L53AI54A. The differences in the scattering profiles, particularly in the region around *q* = 0.28 Å^−1^, are consistent with L53AI54A having a less globular structure than Im7^⁎^. Even though the maximum dimensions of the two proteins are the same, their distance distribution functions (inset in Figure 3(b)) describe this expansion by an increased number of long distance vectors for L53AI54A compared with Im7^⁎^, which is consistent with a redistribution of molecular density away from the centre of gravity of the L53AI54A variant (resulting in a larger radius of gyration (*R*_g_) relative to Im7^⁎^). The *R*_g_ values calculated from the scattering profiles for Im7^⁎^ and the L53AI54A variant of ∼14.6 Å and ∼15.6 Å, respectively, are in reasonable agreement with the *R*_h_ values determined by NMR given the magnitude of the errors involved (Table 1, footnote c). SAXS measurements for the H3G3 variant also indicated that it has an *R*_g_ of ≈19 Å, consistent with the increase in *R*_h_ compared with that of Im7^⁎^ (Table 1). Thus the SIS variants of Im7^⁎^ are globally compact species with structural ensembles having hydrodynamic properties that are expanded to different extents relative to native Im7^⁎^.

### Assignment of the NMR spectra of Im7⁎, L53AI54A and YY

Only two triple resonance experiments (HNCACB, HNCO) were needed to assign the backbone N, HN, C^α^, C^β^, C′ atoms of wild-type Im7^⁎^, reflecting the excellent quality of its HNCACB spectrum and good chemical shift dispersion. Excluding the N-terminal His-tag residues, resonance assignment was completed to 93.0%, 97.6%, 98.9%, 98.8% and 93.0% for each of these atom types, respectively. The peptide resonances of just two residues, Glu2 and Ile44 (non-His-tagged sequence numbering), were not detectable in either ^1^H-^15^N HSQC or triple-resonance spectra under the solution conditions used. By contrast with the excellent spectral quality of Im7^⁎^, L53AI54A and YY required a more comprehensive suite of triple resonance experiments to aid the assignment process, including CBCACONH, HNCA, and HN(CO)CA spectra. Backbone assignments were confirmed with a 750 MHz 3D ^1^H-^1^H-^15^N NOESY-HSQC spectrum (τ_m_ = 100 ms) where sequential NH-NH NOE peak intensity permitted. Overall, assignment statistics for L53AI54A and YY were 75.6% N, 79.3% HN, 85.1% C^α^, 68.3% C^β^, and 75.6% C′ and 80.2% N, 84.1% HN, 86.2% C^α^, 82.1% C^β^, and 79.1% C′, respectively. The missing assignments resulted in the most part from line broadening, presumably reflecting chemical exchange processes in the partially folded ensembles of these proteins.

**Figure 4:**
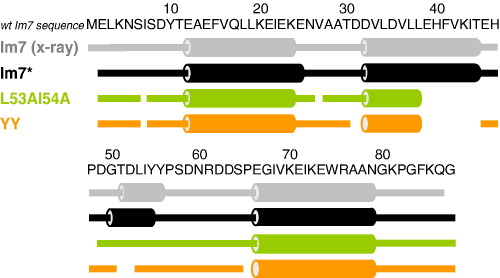
TALOS-derived secondary structure of wild-type Im7^⁎^, L53AI54A and YY aligned with that of the X-ray structure of wild-type Im7 (pdb:1ayi).^20^ Cylinders represent helices and connecting lines represent random coil, as determined by N, C^α^, C^β^ and C′ chemical shifts in TALOS.^31^ Breaks in the secondary structure along the sequence denote residues for which the C^α^ atoms are unassigned and/or the amide resonances could not be detected due to exchange-broadening.

Figure 4 summarises the extent of backbone chemical shift assignments and secondary structure predictions for Im7^⁎^, L53AI54A and YY determined using TALOS.^31^ These data show that for wild-type Im7^⁎^ the solution structure is well represented by the crystal structure of the untagged protein, with the positions of the helices similar to those in the crystal structure. Importantly, comparison of the NMR data for Im7^⁎^ with the corresponding data for Im7 reported by Le Duff *et al*.^29^ shows that the His-tag does not affect the Im7 structured part of Im7^⁎^. The TALOS analysis also showed that L53AI54A and YY have identical secondary structural content to one another, both in length and position. These proteins differ, however, from wild-type Im7^⁎^ in that helical structure in the region corresponding to the native helix III (residues 51–56) is absent. This corroborates observations previously noted by Spence *et al.*^25^ from the results of far-UV CD experiments that the helical content of these trapped intermediates is less than that of native Im7^⁎^ and is consistent with φ-value analysis,^13^ which suggests that helix III is not yet formed in the KIS.

A second, striking difference between the SIS variants L53AI54A and YY and wild-type Im7^⁎^ is the linewidth of peaks resulting from resonances corresponding to the C-terminal region of helix II and the loop between helix II and helix III in the native state. Whilst complete assignment was possible for these regions in wild-type Im7^⁎^, assignments for these residues were not possible for the variant proteins because the resonances were too broad. This presumably reflects exchange-broadening processes that particularly influence resonances in these regions of the variant proteins. Other resonances affected by exchange-broadening that appeared only as weak-intensity peaks in ^1^H-^15^N HSQC spectra involved residues 4–8 (N terminus), 20 (helix I), 25–32 (loop), 49–62 (loop), 66, 68, 72, 73, 76, 79 (helix IV), 84, 86 (C terminus) in L53AI54A and residues 4–10 (N terminus), 20 and 21 (helix I), 30 and 31 (loop), 55 and 56 (loop), 66, 68, 69, 72, 73, 76, 78, 79 (helix IV), and 81 and 84 (C terminus) in YY.

### NMR characterisation of L53AI54A

**Figure 5:**
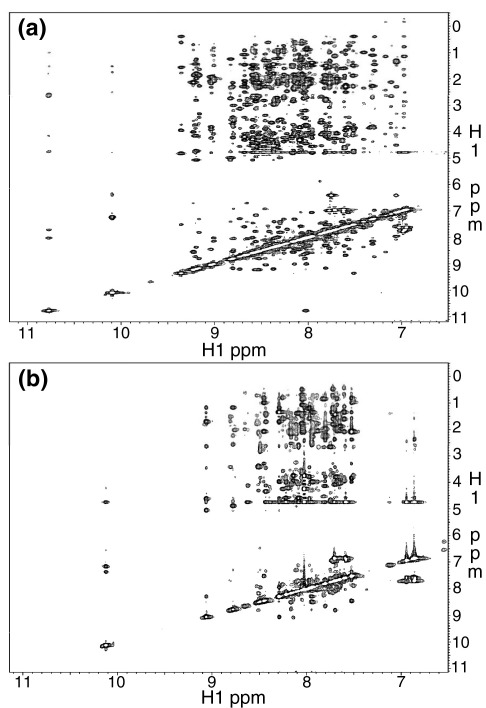
Two-dimensional ^1^H-^1^H projections of 3D ^1^H-^1^H-^15^N NOESY-HSQC data. (a) 500 MHz spectrum of wild-type Im7^⁎^ and (b) 750 MHz spectrum of L53AI54A. Both spectra were acquired with a mixing time of 100 ms at 298 K. Protein concentration was ∼1 mM in 50 mM sodium or potassium phosphate buffer (pH 7.0), 400 mM sodium sulphate, 90% H_2_O/10% ^2^H_2_O and a trace amount of sodium azide. Contour levels are comparable.

Whilst the ^1^H-^1^H-^15^N NOESY-HSQC spectrum of Im7^⁎^ (Figure 5(a)) at 500 MHz contained a large number of NOE cross-peaks, the corresponding spectrum of L53AI54A at 750 MHz contained far fewer peaks (compare Figure 5(a) and (b)). In particular, both short-range and long-range NOEs involving side-chain resonances were severely broadened, whilst NH-NH NOEs were also weak. Detailed analysis of the L53AI54A spectrum shows that although most *d*_NN_(*i,i* + 1) and some *d*_NN_(*i,i* + 2) NOEs for residues of helix I were strong, the *d*_NN_(*i,i* + 1) NOEs for helix II were only of medium intensity and there were no *d*_NN_(*i,i* + 2) NOEs between residues in this helix. For helix IV most *d*_NN_(*i,i* + 1) NOEs were weak and there were no *d*_NN_(*i,i* + 2) NOEs. The data thus suggest that whilst elements of secondary structure involving helices I, part of II, and IV are formed in the SIS, the orientation of these helices and the packing of side-chains is still relatively fluid, with the different arrangements of the helices in exchange on the NMR timescale. Consistent with a dynamic structural ensemble, it was not possible to determine rates of native-state hydrogen exchange for the peptide NH groups in L53AI54A or YY since all the amides exchanged too rapidly with solvent to detect any signals within the dead-time of the experiment (2 min).

### NMR relaxation studies of Im7⁎ and L53AI54A

**Figure 6:**
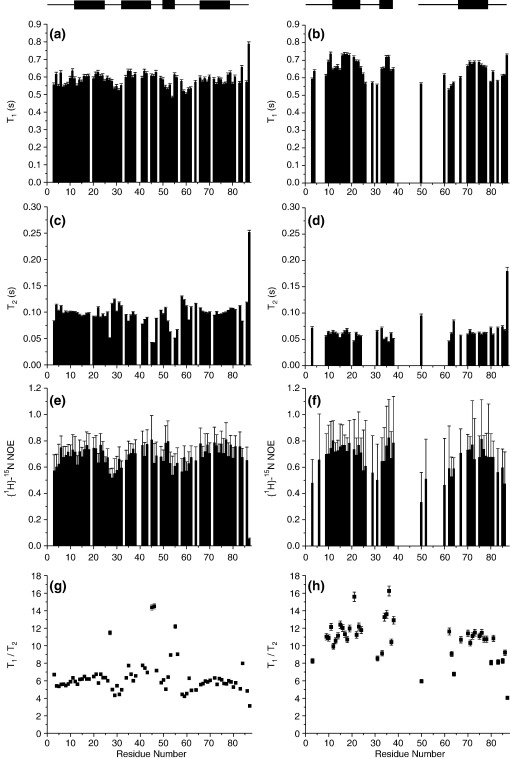
Comparison of backbone ^15^N relaxation parameters determined for wild-type Im7^⁎^ (left panels) and L53AI54A (right panels) at 600 MHz, 298 K. (a) and (b) *T*_1_ relaxation times; (c) and (d) *T*_2_ relaxation times; (e) and (f) {^1^H}-^15^N heteronuclear NOE; (g) and (h) *T*_1_/*T*_2_ ratio. The average errors in the relaxation parameters for wild-type Im7^⁎^ and L53AI54A, respectively, are 1.6% and 1.2% (*T*_1_), 1.3% and 3.2% (*T*_2_), 14.1% and 32.8% (NOE), 2.0% and 3.4% (*T*_1_/*T*_2_ ratio). Error bars are displayed although in some cases the error bar is smaller than the size of the symbols used. Residues in Im7^⁎^ for which no data are shown results from very weak signal intensity (residue 54), severe resonance overlap (residues 19, 33, 39, 40, 63, 71, 85), undetectable resonances (residues 1, 2, 44), or residues that are proline (residues 48, 57, 65, 82). Likewise, in L53AI54A no data results from very weak signal intensity (residues 6, 8, 30, 51, 52, 53, 55, 56, 59, 60, 66, 68, 79), severe resonance overlap (residues 5, 20, 58, 61, 69, 74, 84), undetectable resonances (residues 1, 2, 7, 27, 28, 32, 39–49, 54), or residues that are proline (residues 48, 57, 65, 82). The secondary structure of wild-type Im7^⁎^ and L53AI54A as predicted by TALOS^31^ is depicted above the left and right columns of the data, respectively. Protein concentration was ∼1 mM in 50 mM sodium/potassium phosphate buffer (pH 7.0), 400 mM sodium sulphate, 90% H_2_O/10% ^2^H_2_O and a trace amount of sodium azide.

In order to investigate the dynamical properties of L53AI54A in more detail, ^15^N relaxation data were collected for 73 and 72 of the 87 Im7 residues of Im7^⁎^ at 60.72 MHz (for *T*_1_ and {^1^H}-^15^N NOE measurements and *T*_2_, respectively) and for 47, 42 and 49 residues of L53AI54A (for *T*_1_, *T*_2_ and {^1^H}-^15^N NOE measurements, respectively) at 60.78 MHz (Figure 6). Residues were excluded either because they were proline, unassigned, or because their resonances were too weak or overlapped with others for accurate determination of relaxation data. For wild-type Im7^⁎^ the resulting data are consistent with a well-structured globular protein (Figure 6(a), (c) and (e)). Consistent with this, there is only a small variation in ^15^N *T*_1_ and *T*_2_ relaxation times and {^1^H}-^15^N NOE values along the sequence, with only the C-terminal residue (Gly87) and residues in inter-helix loop regions, the C terminus of helix II, and helix III showing enhanced dynamic properties (Figure 6(a), (c) and (e)). This is best brought out by the sequence variation in the *T*_1_/*T*_2_ ratios for backbone ^15^N resonances (Figure 6(g)). For L53AI54A, by contrast, the average *T*_1_ is higher, the average *T*_2_ significantly lower and the average NOE reduced compared with wild-type Im7^⁎^, confirming that the trapped intermediate ensemble exhibits dramatically different conformational dynamics from those of Im7^⁎^ (Figure 6(b), (d), (f) and (h)). The observed differences in ^15^N backbone relaxation parameters for Im7^⁎^ and L53AI54A indicate that the NH resonances of the latter are influenced by local motions as well as by global tumbling. Such increased conformational dynamics of L53AI54A rules out analysis of its dynamic properties using the model-free formalism, since the assumption of a single overall correlation time required for such an analysis is not valid. This is because one of the consequences of substantial local dynamics is that the effect of internal motions can no longer be separated from those of molecular tumbling.^32^ By contrast with this approach, reduced spectral density mapping does not require assumptions about the nature of internal motions within the protein to determine the reduced spectral density functions *J*(0), *J*(ω_N_), and *J*(0.87 ω_H_).^32–34^ We thus used this approach to investigate the conformational dynamics of L53AI54A in more detail. The magnitudes of the *J*(0), J(ω_N_), and *J*(0.87 ω_H_) functions are sensitive to motions at different frequencies with *J*(0) reflecting slow internal motions on the millisecond to microsecond time scale as well as slow global rotational diffusion, *J*(0.87 ω_H_) reporting on the presence of internal motions on the picosecond timescale, and *J*(ω_N_) lying between these extremes.

**Figure 7:**
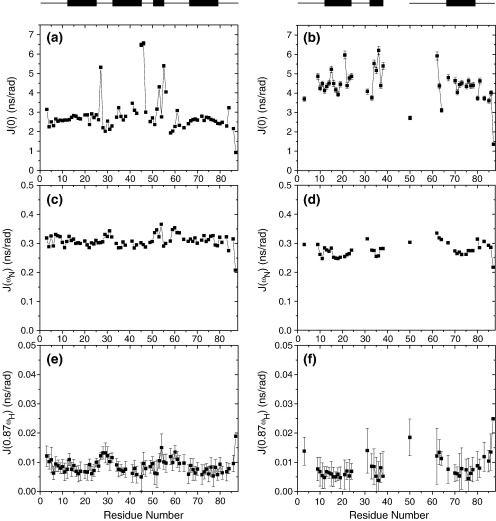
Reduced spectral density functions, *J*(0) ((a) and (b)), *J*(ω_N_) ((c) and (d)), and *J*(0.87 ω_H_) ((e) and (f)) for wild-type Im7^⁎^ (left panels) and L53AI54A (right panels) derived from *R*_1_, *R*_2_ and NOE relaxation data at 600 MHz, 298 . The secondary structure of wild-type Im7^⁎^ and L53AI54A as predicted using TALOS^31^ is depicted above each column of data for reference. The average errors for wild-type Im7^⁎^ and L53AI54A, respectively, are 1.4% and 3.3% (*J*(0)), 0.2% and 0.3% (*J*(ω_N_)), 33.0% and 64.4% (*J*(0.87 ω_H_). Error bars are displayed although in some cases the error bar is smaller than the size of the symbols used. Residues in Im7^⁎^ for which no data are shown results from very weak signal intensity (residue 54), severe resonance overlap (residues 19, 33, 39, 40, 63, 71, 85), undetectable resonances (residues 1, 2, 44), or residues that are proline (residues 48, 57, 65, 82). Likewise, in L53AI54A no data results from very weak signal intensity (residues 6, 8, 30, 51, 52, 53, 55, 56, 59, 60, 66, 68, 79), severe resonance overlap (residues 5, 20, 58, 61, 69, 74, 84), undetectable resonances (residues 1, 2, 7, 27, 28, 32, 39–49, 54), or residues that are proline (residues 48, 57, 65, 82).

**Figure 8:**
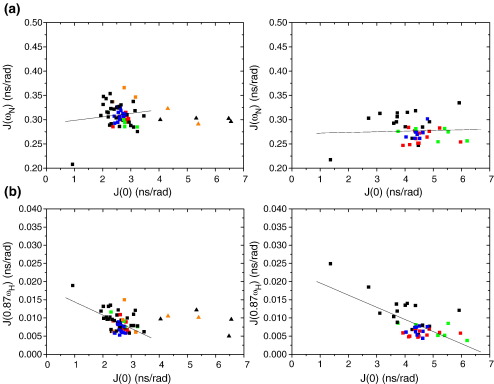
Plots of *J*(0) against (a) *J*(ω_N_) and (b) *J*(0.87 ω_H_) for residues of Im7^⁎^ (left panels) and L53AI54A (right panels), respectively. Residues located in helices are colour-coded as follows: helix I (red), helix II (green), helix III (orange), helix IV (blue). Black symbols depict non-helical residues. Continuous lines represent the line of best fit through the data and were calculated using a linear least-squares fit. ▴ symbols indicate residues in Im7^⁎^ that experience conformational exchange and were not included in the linear least-squares fit of the Im7^⁎^ data. The slopes for the line of best fits in (a) are 0.0073 ± 0.007 (Im7^⁎^) and 0.0014 ± 0.004 (L53AI54A), with correlation coefficient, *R*, values of 0.1207 and 0.0487, respectively; and in (b) are −0.0036 ± (7.16 × 10^−4^) (Im7^⁎^) and −0.0033 ± (5.69 × 10^−4^) (L53AI54A), with correlation coefficient, *R*, values of −0.5329 and −0.6797, respectively.

Plots of *J*(0), *J*(ω_N_) and *J*(0.87 ω_H_) as a function of sequence number are shown in Figure 7 for wild-type Im7^⁎^ and L53AI54A. As commonly observed,^32–34^ the reduced spectral density functions *J*(0), *J*(ω_N_) and *J*(0.87 ω_H_) for both proteins reflect the variations in 1/*T*_2_, 1/*T*_1_ and the {^1^H}-^15^N NOE, respectively. For wild-type Im7^⁎^, the profiles of observed spectral densities *J*(ω_N_) and *J*(0.87 ω_H_) are typical of globular proteins, showing low values for residues located in well-ordered regions of the protein and increased values for residues undergoing high frequency motions. Lefèvre *et al.*^35^ and Viles *et al.*^36^ showed that plots of *J*(0) against *J*(ω_N_) and *J*(0.87 ω_H_), which should be linearly correlated, can be informative about the internal motions of a protein and such plots for Im7^⁎^ are given in Figure 8. In these plots residues in the helices tend to fall into the regions of high *J*(0) and relatively low *J*(ω_N_) and *J*(0.87 ω_H_), while the non-helical regions tend to cluster in regions of smaller *J*(0) and greater *J*(ω_N_) and *J*(0.87 ω_H_), consistent with the helical core of the protein having a correlation time influenced by the overall tumbling rate more than local dynamics, and the non-helical regions experiencing a greater effect from local dynamics. Thus, the high frequency motions (nanoseconds–picoseconds) observed in wild-type Im7^⁎^ (Figure 7(c) and (e)) coincide well with loop regions; in particular, the loop between helix I and helix II (residues 26–31) and the loop between helix III and helix IV (residues 57–65). However, residues located in helix III behave differently from those in helices I, II and IV (Figures 7 and 8), exhibiting increased *J*(ω_N_) and *J*(0.87 ω_H_) values. Additionally, the *J*(0) values for residues 53, 55 and 56 (which lie in the native helix III) are larger than one standard deviation above the mean, and such pronounced values are typical of residues for which a chemical exchange term is needed to describe the short *T*_2_ value observed. Thus, even for wild-type Im7^⁎^ chemical exchange on the milliseconds–microseconds timescale affects resonances of residues in helix III. Elevated *J*(0) values occur for three other residues across the sequence (Figure 7(a)), located at the C terminus of helix II and in the following loop (45,46) and for residue 27 (loop between helix I and helix II), suggesting a significant contribution of slow internal motions to the conformational dynamics of wild-type Im7^⁎^. The NMR relaxation data allow the overall rotational correlation time, τ_c_, to be estimated. Using the approximation τ_c_∼5/2 〈*J*(0)〉,^32^ and excluding residues with *J*(0) values larger than one standard deviation above or below the mean, a value for τ_c_ of 6.55(±0.8) ns for Im7^⁎^ is obtained. This is in excellent agreement with the value of 6.52 ns obtained from the *R*_h_ of Im7^⁎^ determined by SAXS (19.3 Å) assuming a spherical structure, and in good agreement with the τ_c_ of the related protein Im9 of ∼7 ns determined from ^15^N relaxation data.^37^

For those residues in L53AI54A for which it was possible to obtain relaxation data, the overall trend in the reduced spectral density function at the three frequencies *J*(0), *J*(ω_N_) and *J*(0.87 ω_H_) is similar to that of wild-type Im7^⁎^ (Figures 7 and 8). What differs between the two proteins, however, is the spread of the values of the reduced spectral density functions, which is significantly larger in L53AI54A. This is especially apparent at the *J*(0) frequency (Figure 7(b)), for which the standard deviation from the mean *J*(0) value is 0.85 ns rad^−1^ over all residues measured in L53AI54A compared with 0.37 ns rad^−1^ over the same residues in wild-type Im7^⁎^. Another striking difference between the spectral densities of the two proteins is that resonances of residues observed to undergo chemical exchange in wild-type Im7^⁎^ (residues 27, 45, 46, 53, 55 and 56) are not detected (27, 45, 46) or are much weaker (53, 55, 56) in spectra of L53AI54A, suggesting that these resonances are considerably more exchange-broadened in L53AI54A than in wild-type Im7^⁎^. Furthermore, the value of *J*(0) in L53AI54A is raised across the sequence compared with wild-type Im7^⁎^ with a mean of 4.36 ns rad^−1^ in L53AI54A (excluding residues more than one standard deviation from the mean) compared with 2.62 ns rad^−1^ over the same residues in wild-type Im7^⁎^. Since L53AI54A has only a slightly greater *R*_h_ than Im7^⁎^ (Table 1) this increase in *J*(0) cannot be due to an increase in the global rotational tumbling time and thus must arise from significant chemical exchange on the milliseconds–microseconds time scale that affects much of the sequence of L53AI54A.

## Discussion

### The conformational ensemble and dynamics of the SIS

Whilst previous analyses have suggested that the trapped intermediate SIS variants of Im7^⁎^ are highly helical species,^25^ the NMR analysis presented here has provided much more detailed insight into their structure and dynamics. Analysis of backbone NMR chemical shifts of L53AI54A and YY demonstrates that these SIS variants contain three helices in common with wild-type Im7^⁎^, with helices I and IV being native-like and helix II at least partially formed. Consistent with previous analysis which demonstrated that the trapped intermediate contains substantial tertiary structure that is cooperatively stabilised,^25^ measurement of the *R*_h_ and SAXS data (Figure 3) of L53AI54A also points to a globular structure, albeit less so in the variants compared with native wild-type Im7^⁎^. The data suggest, therefore, that L53AI54A has a three-helical structure consistent with φ-value analysis of the KIS of Im7^⁎^^13^ and molecular dynamics of the NH exchange competent EIS.^24^ However, by contrast with the well-formed secondary structure in this variant (Figure 4), the NMR relaxation studies (Figures 6–8), NH exchange data and paucity of NOE peaks in NOESY spectra (Figure 5) suggest that the hydrophobic core of L53AI54A remains relatively fluid, with conformational exchange occurring between different species within the SIS ensemble. In native Im7,^20^ residues in helix III lie in close proximity to residues at the C-terminal end of helix II and whilst helix III is not formed in the EIS and helix II is, the C-terminal end of helix II has NH exchange rates greater than those predicted from Δ*G*_NU_, suggesting that local motions superimposed on global unfolding affect exchange rates for residues in this region. The lack of assignments for residues lying toward the C-terminal end of helix II in L53AI54A and YY (Figure 4) reflects chemical exchange broadening, indicating that, as in the EIS, the C-terminal end of helix II in the SIS has enhanced dynamic features compared with helices I and IV. Such exchange may rationalise the relatively low φ-values observed for the KIS (of the 25 residues measured, 17 have φ-values less than 0.7, and 13 of these lie in helices I, II and IV in native Im7^13^) and may also account for the observation that although many of the peptide NH hydrogen atoms of the helices in Im7^⁎^ exchange for deuterons with a free energy greater than Δ*G*_IN,_ the majority exchange with a free energy lower than that of the global unfolding equilibrium.^23^

H3G3 and H3G6 have significantly different NMR characteristics compared with L53AI54A and YY, as revealed by their ^1^H-^15^N HSQC spectra (Figure 2) and *R*_h_ values (Table 1), and yet resemble L53AI54A and YY in their helical content and stabilities, as shown by Spence *et al.*^25^ Thus it is likely that these are also three-helix bundles with helices I, II and IV formed to similar extents as in L53AI54A and YY, but with exchange within their conformational ensembles leading to broader NMR signals. The cause of the difference in spectral properties between H3G3/H3G6 and L53AI54A/YY must be the presence of one or both of the tyrosine residues at positions 55 and 56 of L53AI54A/YY, since these are the only sequence differences between YY and H3G6 (Table 1). Moreover, unlike L53AI54A and YY, which resemble the Im7^⁎^ KIS in being hyper-fluorescent, H3G3 and H3G6 are not hyper-fluorescent.^25^ The hyper-fluorescence therefore appears to result from an alteration in the environment of Trp75 in the core of the protein through interactions (either directly or indirectly) with Tyr55 and/or Tyr56. This is consistent with UV-RR data^26^ and with the structure determination of the EIS of Im7^⁎^ reported by Gsponer *et al.,*^24^ which suggest a perturbed environment of these Tyr residues relative to wild-type Im7^⁎^. In the EIS conformational ensemble, Tyr55 and Tyr56 are predicted to be significantly closer to Trp75 than they are in the native structure, suggesting that tyrosine-tryptophan energy transfer contributes to the hyper-fluorescence of the intermediate.^24^ Therefore we suggest that for L53AI54A and YY one or both of Tyr55/Tyr56 interact with the three-helix core thereby reducing its dynamics compared with H3G3 and H3G6.

In summary, the NMR data presented here indicate that the SIS variants have an overall rather native-like topology comprising three of the native helices stacked around a hydrophobic core that is conformationally dynamic. These conformational dynamics preclude determination of a unique structure for this ensemble. By contrast, NMR structures of several protein folding intermediate mimics stabilised by site-directed mutagenesis have recently been reported, including the Leu16Ala variant of engrailed homeodomain (En-HD)^18^ and a partially unfolded form of the four-helix bundle protein apocytochrome *b*_562_ (Rd-apocyt).^9^ In these cases, NOESY analyses were central to the structure determinations. For L53AI54A, by contrast, exchange between members of the conformational ensemble acts as a leakage mechanism for inter-residue NOEs, with the effect that direct structural analysis was not possible. Presumably, such a situation does not pertain to En-HD and Rd-apocyt, as indicated by their NMR relaxation parameters. From the *J*(0) data reported by Religa *et al.* for the Leu16Ala variant of En-HD^18^ we estimate that its τ_c_ is ∼5.7 ns, which is in line with its mass,^38^ indicating that chemical exchange terms do not contribute to its resonance linewidths, by contrast with the dynamic properties of L53AI54A. Though reduced spectral density mapping of Rd-apocyt has not been reported, backbone ^15^N relaxation parameters suggest that this protein contains a flexible part with relaxation characteristics similar to those of L53AI54A, for which Feng *et al.*^9^ did not determine a structure, and a structured part with ^15^N *T*_2_ values lacking contributing chemical exchange terms and yielding sufficient NOEs to allow its structure to be determined.^9^ Thus, whereas the stabilised intermediate of En-HD shows little or no interconversion between alternative conformers on the milliseconds timescale and the structured domain of Rd-apocyt appears to resemble a highly native-like state, the SIS of Im7^⁎^ is more akin to a classical intermediate state, in which multiple conformational species in dynamic equilibrium co-exist within an ensemble of structures that contain a highly native-like content of secondary structure, but in which the packing of the helices has yet to become rigidly fixed in space.

### Implication for the folding mechanism of Im7⁎

Together with previously reported φ-value analyses of the Im7^⁎^ KIS and TS2,^13^ the data presented here provide direct insights into the conformational properties of the folding intermediate of Im7^⁎^ and suggest implications for how this species is reorganised as the native state develops. Previous φ-value analysis of the KIS,^13^ now supported by the TALOS analysis of the chemical shifts of the SIS variants L53AI54A and YY (Figure 4), suggests that these intermediates have a common architecture consisting of a three-helical structure comprising helices I, II and IV. Despite possessing similarities in their secondary structure content, however, we show that the hydrophobic core of these proteins is not yet well formed, in that fixed tertiary interactions have not yet developed, resulting in a broad ensemble of similar structures that are in exchange on the NMR timescale. The comparison of NMR and biophysical data for H3G3/H3G6 and L53AI54A/YY suggests that one or more residues in the region of the protein from residue 50–60 may interact with the three-helix bundle core before helix III is formed. In this respect then, the three helix core acts as a template to assist in formation of helix III, the shorter side-chains at positions 53 and 54 of L53AI54A compared with those in the wild-type protein disfavouring formation of the key stabilising interactions essential for formation of a docked, and therefore uniquely structured helix III, effectively trapping the protein in the intermediate state. φ-value analysis of the subsequent rate-limiting transition state ensemble for native state formation, TS2 (Figure 1(b)) during Im7^⁎^ folding shows that Thr51 and Leu53, both of which lie in the native helix III in Im7^⁎^ (Figure 1(a)), have φ-values close to zero, whilst Ile54 has a φ-value of 0.16 when it is replaced by valine, suggesting that this residue may be weakly interacting in TS2. We therefore suggest that TS2 is the state in which conformational rearrangement of the core occurs to optimise interactions with residues 50–56 so that helix III can begin to form. As it does so molecules descend to the native state and the formation of helical structure corresponding to the native helix III takes place. As this is not possible for L53AI54A and YY, molecules that approach TS2 retreat to the KIS, effectively trapping the protein in an inactive, three-helical state. The docking of residues in the region spanning the native helix III to the three-helix core raises the energy of the conformational ensemble from that of the KIS to that of TS2 because the conformational entropy of the protein is reduced by the docking and this is not off-set immediately by hydrogen-bond formation within helix III, which occurs on the downward slope from TS2 to the native state. Thus, energetic interactions by helix III residues are the key to formation of the native four-helix bundle structure of Im7 as well as formation of the DNase-Im7 complex,^19^ which is the physiological function of Im7.

## Materials and Methods

### Sample preparation

All quoted pH values are direct meter readings uncorrected for any isotope effects. Im7 was over-expressed using plasmid pRJ347, based on the expression vector pTrc99A (Pharmacia) and purified using anion exchange column chromatography followed by S75 gel filtration column chromatography with minor modifications from the method described.^39^ Im7^⁎^ and its SIS variants were prepared as described.^25^ ^15^N and ^13^C/^15^N labelling was carried out by growing cells containing the relevant plasmid in M9 media enriched with ^15^NH_4_Cl (1 g/l) and [^13^C_6_]glucose (4 g/l)/^15^NH_4_Cl (1 g/l), respectively, 100× MEM vitamin solution (10 ml/l), 0.01 M FeCl_3_ (1 ml/l), 100 mg/ml carbenicillin stock (1 ml/l), 1% (w/v) thiamine solution (0.2 ml/l), 1 M MgSO_4_ (2 ml/l), and 100 mM CaCl_2_ (2 ml/l). NMR experiments were measured on samples containing ∼1 mM protein (^15^N or ^13^C/^15^N-labelled) in 600 μl of buffer (pH 7.0) made up of 50 mM KH_2_PO_4_ or NaH_2_PO_4_, 400 mM Na_2_SO_4_, 10% (v/v) ^2^H_2_O and a trace amount of NaN_3_. Samples for hydrodynamic radius measurements were made up in buffer containing 100% ^2^H_2_O (all other buffer components as above, pH 7.0) and also contained 20 μl of 1% (w/v) 1,4-dioxane in ^2^H_2_O.

### NMR spectroscopy

All NMR experiments were performed at 298 K. With the exception of a 3D ^1^H-^1^H-^15^N NOESY-HSQC spectrum (τ_m_ = 100 ms) measured on a Varian Inova 750 MHz spectrometer, all spectra were acquired with Varian Unity Inova 500 or 600 MHz spectrometers equipped with 5 mm triple resonance pulsed field gradient probes, operating at ^1^H Larmor frequencies of 499.82 MHz and 599.16 or 599.80 MHz, respectively, and ^15^N Larmor frequencies of 50.65 MHz and 60.72 or 60.78 MHz, respectively. 750 MHz data were measured on a similarly equipped spectrometer, operating at a ^1^H Larmor frequency of 749.83 MHz and a ^15^N Larmor frequency of 75.99 MHz. Pulse sequences from the Varian (CA, USA) “Protein Pack” suite of experiments were used for standard triple resonance measurements,^40^ 3D NOESY-HSQC and ^15^N relaxation data collection. A τ_m_ of 100 ms was used for all NOESY spectra. Proton chemical shifts were referenced against external 2,2-(dimethylsilyl)propanesulfonic acid (DSS) while nitrogen and carbon chemical shifts were referenced indirectly to DSS using the absolute frequency ratios.^41^ Owing to the high salt concentration present in all samples, longer ^1^H 90° pulse widths of ∼8.3 μs (500 MHz) and ∼9.3 μs (600 MHz) were required, compared with ∼6.1 μs (500 MHz) and ∼6.9 μs (600 MHz) in the absence of sodium sulphate. ^15^N pulse widths were unaffected by the high salt concentration. A recycle delay of 1 s was used in all experiments. Other data acquisition parameters are given in Table 1 of the Supplementary Material. One-dimensional spectra were processed and analysed using Varian VNMR software, while multidimensional data were processed using NMRPipe.^42^ Prior to the Fourier transformation of all multidimensional spectra, a cosine-bell window function was applied to each dimension. Linear prediction was performed in all indirect dimensions using the automated order, followed by zero-filling (in all dimensions) to round the final number of data points to the power of 2. Data sets were analysed using NMRView version 5.^43^

Chemical shifts of C^α^, C^β^, C′ and N atoms were used as input for TALOS^31^ to predict the backbone angles, phi and psi. The RAMA component of the program was then used to display the Ramachandran map and identify the regions occupied by the predicted backbone angles, thereby predicting secondary structure. Secondary structure was only accepted for residues defined as having good predictions as defined by Cornilescu *et al.*^31^

^15^N *T*_1_, ^15^N *T*_2_ and {^1^H}-^15^N NOE data were collected for ^15^N-labelled wild-type Im7^⁎^ and L53AI54A at 600 MHz using the procedures described by Kay *et al.*^44^ and Farrow *et al*.^45^ Spectra of wild-type Im7^⁎^ were recorded as matrices of 512 × 128 complex data points with spectral widths of 8000 Hz (^1^H) and 1680 Hz (^15^N) using 32 scans for *T*_1_, *T*_2_ data and 64 scans for {^1^H}-^15^N NOE data. Relaxation time spectra of L53AI54A were recorded as matrices of 1024 × 256 complex data points with spectral widths of 8000 Hz (^1^H) and 1650 Hz (^15^N) using 40 scans per *t*_1_ increment. {^1^H}-^15^N NOE data were collected as matrices of 1024 × 204 complex data points with the same spectral widths as for *T*_1_ and *T*_2_ spectra. Eighty scans were measured per *t*_1_ increment. For wild-type Im7^⁎^, *T*_1_ data were acquired with relaxation delays of 10, 50, 80, 200, 500, 750 ms, 1 and 2 s, duplicating the experiments with 10, 200 and 500 ms delays for the determination of peak height uncertainties.^46^ *T*_2_ experiments were measured with relaxation delays of 10, 30, 50, 70, 110, 150 and 250 ms, with the experiments at 10, 50 and 150 ms repeated. For L53AI54A, *T*_1_ data were acquired with relaxation delays of 11, 55, 111, 222, 388, 555, 777 and 999 ms with the experiments at 55 and 388 ms repeated. *T*_2_ experiments were measured with relaxation delays of 17, 33, 50, 66, 83, 99, 116 and 132 ms, with the experiments at 33 and 116 ms repeated. Recycle delays of 4 s (wild-type Im7^⁎^) and 1 s (L53AI54A) were used for the measurement of *T*_1_ and *T*_2_ data. All *T*_1_ and *T*_2_ data were acquired in an interleaved manner, to minimise the effects of sample heating. For steady-state NOE determination, two spectra were acquired, with and without proton saturation during the 5 s relaxation delay. Proton saturation was achieved with a pulse train of 120° pulses every 5 ms for 3 s. Triplicate sets of interleaved saturated/unsaturated experiments were measured for wild-type Im7^⁎^ to determine the uncertainties of the NOE values, taken to be the standard deviation of the average NOE determined from the three repeat experiments. Only one set of saturated/unsaturated experiments was recorded for L53AI54A so uncertainties in the peak heights of NOE spectra of L53AI54A were given by the standard deviation of baseplane noise in the spectra as described by Skelton *et al*.^47^ Uncertainties in the NOE values were obtained by propagating the uncertainties in the peak heights.^48^ NOE values were determined as the ratio of the peak heights with and without proton saturation. Relaxation times were determined by fitting peak heights as a function of the relaxation delay to a two-parameter single exponential decay using CurveFit (A.G. Palmer, Columbia University). Three-parameter curve-fitting^49^ for weak intensity peaks was investigated but in all cases two-parameter fits were superior. Uncertainties in the relaxation times were taken to be the standard errors of the fitted parameters. Relaxation times and heteronuclear NOEs were obtained using programs generously provided by Professor Arthur G. Palmer†. All peak heights were measured using the non-linear spectral lineshape modelling (nlinLS) routine in NMRPipe.^42^ Spectra were processed using a Lorentzian-Gaussian window function for apodization in ω2 (inverse exponential width of 8 Hz, Gaussian width of 10 Hz), while in ω1 the same Lorentzian-Gaussian transformation followed by a cosine-bell was used. Linear prediction was applied to the indirect dimension of all spectra of wild-type Im7^⁎^ and the NOE spectra of L53AI54A. Residues were excluded from analysis where resonance overlap was too severe or peak intensity too weak to reliably determine the relaxation parameters. Reduced spectral density mapping was performed using a program kindly provided by Dr Mike Osborne (University of Montreal, Canada) to analyse relaxation data using the procedure described by Farrow *et al.*^50^ The spectral density functions *J*(0), *J*(ω_N_) and *J*(0.87 ω_H_) were obtained by assuming that the variation in *J*(ω) is relatively constant between *J*(ω_H_ + ω_N_) and *J*(ω_H_–ω_N_). Chemical shift anisotropy was taken to be −170 ppm and the N-H bond length was 1.02 Å.

Hydrodynamic radii were measured by the approach described by Wilkins *et al.*^28^ essentially as described,^37^ except that the water_sLED_fm_v2_500 pulse sequence based on that described by Altieri *et al.*^51^ was employed, which incorporates WATERGATE solvent suppression. Each experiment was acquired in triplicate for error analysis and with 256 scans. Gradient strengths between 1.7 and 32.2 G cm^−1^ at 298 K were used. Owing to severe resonance overlap and line-broadening in the Im7^⁎^ variant proteins, it was only possible to measure accurate peak heights for the more intense His-tag resonances in H3G3 and H3G6. For L53AI54A and YY, two His-tag resonances and one other reasonably well-isolated aromatic resonance were measured. For wild-type Im7^⁎^, an additional aromatic resonance was also measured. In each sample, the decay of the dioxane peak with gradient strength was used as the reference, with *R*_h_^ref^ = 2.12 Å.^28^

### Solution X-ray scattering data collection and analysis

X-ray scattering experiments were carried out at station 2.1 of the Daresbury Synchrotron Radiation Source^52^ using a 200 mm × 200 mm position-sensitive multi-wire proportional counter operated at 512 × 512 pixels.^53^ Scattering data from buffer and protein samples (with concentrations between 1 and 10 mg/ml in 50 mM potassium phosphate buffer (pH 7.0) and 400 mM sodium sulphate (His-tagged protein only)) were collected at 10 °C at a sample-to-detector distance of 1 m allowing a momentum transfer of 0.04 Å^−1^ < *q* < 0.78 Å^−1^ to be measured (with *q* = 4π sinθ/ λ, where 2θ is the scattering angle and λ the X-ray wavelength of 1.54 Å). The systematic data reduction included radial integration of the two-dimensional images, normalisation of the subsequent one-dimensional data to the intensity of the transmitted beam, correction for detector artefacts and subtraction of background scattering from the buffer. Silver behenate powder was used to calibrate the *q*-range (based on a diffraction spacing of 58.38 Å). The distance distribution function *p*(*r*) and the radius of gyration *R*_g_ were evaluated with the indirect Fourier transform program GNOM,^54^ which also leads to a reliable estimate of the maximum particle dimension *D*_max_, the value of *r* at which *p*(*r*) goes down to zero. The program CRYSOL^55^ was used for the scattering pattern simulation from crystal structure data. This analysis gives a discrepancy factor or goodness-of-fit value (χ) as a measure of how well the structural model fits the experimental data. A good agreement between experiment and simulation is generally obtained for χ-values smaller than 2 to 3.

### Data bank accession codes

Sequence-specific assignments have been deposited in BioMagResBank (entries 7316, 7317 and 7318).

## Figures and Tables

**Table 1:**
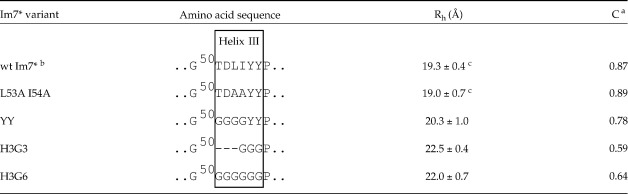
Hydrodynamic radii (*R*_h_) and compaction factors (*C*) for wild-type Im7^⁎^ and the SIS variants L53AI54A, YY, H3G3 and H3G6 determined at 600 MHz, 25 °C

^**a**^Compaction factors calculated using the equation *C* = (*R*_D_^h^–*R*_h_) / (*R*_D_^h^–*R*_N_^h^) as described by Wilkins *et al.*^28^, where *R*_N_^h^ and *R*_D_^h^ are the predicted values of hydrodynamic radii for the native and fully denatured states, respectively, and *R*_h_ is the experimental hydrodynamic radius. ^**b**^ The *R*_h_ and *C* of Im7 are 17.3(±0.8) Å and 1.04, respectively.^29^ ^**c**^*R*_h_ values calculated from SAXS measurements of *R*_g_ at 10 °C using the relationship *R*_g_ = (3/5)^1/2^*R*_h_^30^ were 18.8 Å and 20.1 Å, respectively, for Im7^⁎^ and L53AI54A.

## Footnotes

† http://tinyurl.com/9oysy
